# Computational pathology in the identification of HER2-low breast cancer: Opportunities and challenges

**DOI:** 10.1016/j.jpi.2023.100343

**Published:** 2023-11-04

**Authors:** Marie Brevet, Zaibo Li, Anil Parwani

**Affiliations:** a Cypath-RB, Lyon, France; b Department of Pathology, The Ohio State University, Columbus, OH, USA

**Keywords:** HER2, breast carcinoma, computational pathology, HER2-low, immunohistochemistry, digital pathology

## Abstract

For the past 2 decades, pathologists have been accustomed to reporting the HER2 status of breast cancer as either positive or negative, based on HER2 IHC. Today, however, there is a clinical imperative to employ a 3-tier approach to interpreting HER2 IHC that can also identify tumours categorised as HER2-low. Meeting this need for a finer degree of discrimination may be challenging, and in this article, we consider the potential for the integration of computational approaches to support pathologists in achieving accurate and reproducible HER2 IHC scoring as well as outlining some of the practicalities involved.

## The clinical rationale for identifying HER2-low breast cancer

Human epidermal growth factor receptor 2 (HER2) is overexpressed due to amplification of the *HER2* gene in around 15% of breast carcinomas.^1^^,^^2^ Tumour cell HER2 expression is assessed in patients with breast cancer to select those who are eligible for targeted anti-HER2 therapies.^1^

In routine diagnostic practice, immunohistochemistry (IHC) is used to determine the level of HER2 protein expression, with or without reflex *in situ* hybridisation (ISH) to detect *HER*2 gene amplification.^1^, ^2^, ^3^ Based on IHC, the level of HER2 protein expression is assigned a discrete score of 0, 1+, 2+, or 3+. According to current HER2 testing guidelines, a tumour is considered HER2-positive if the IHC score is 3+ or if it is 2+ and there is evidence of *HER*2 gene amplification via ISH.^4^ Tumours with an IHC score of 2+ but without evidence of *HER*2 gene amplification are classified as HER2-negative, along with those that have IHC scores of 1+ or 0.

Over the past several years, clinical outcomes for patients with HER2-positive breast cancer have been significantly improved through the use of anti-HER2 monoclonal antibodies.^2^^,^^5^ Meanwhile, a lack of benefit from anti-HER2 monoclonal antibodies has meant that patients with HER2-negative disease have not been eligible for such treatment. HER2-negative patients include those with triple-negative breast cancer (oestrogen receptor (ER)-negative, progesterone receptor (PR)-negative, and HER2-negative), which is associated with an aggressive disease course and particularly poor outcomes.^6^

Recently, however, HER2-based antibody–drug conjugates (ADCs) have been developed that, unlike traditional anti-HER2 monoclonal antibodies, can be effective in tumours that lack strong oncogenic addiction to *HER2* but have lower levels of HER2 expression.^7^ While traditional anti-HER2 monoclonal antibodies act by blocking aberrant HER2 signalling via dimerisation inhibition, HER2 internalisation, and potent antibody-dependent cellular cytotoxicity, ADCs are designed to target tumour cells and cause cell death by delivering cytotoxic chemotherapy directly.

Patients with tumours that have HER2 IHC scores of 1+ or 2+ without *HER*2 gene amplification have been shown to benefit from treatment with the HER2-based ADC trastuzumab deruxtecan.^8^^,^^9^ Such patients are classified as having HER2-low breast cancer, and they comprise around 50% of all breast cancer cases.^2^^,^^10^

The ability to identify HER2-low breast cancer is critical for a patient population that would otherwise not be treated with anti-HER2 therapy.

## Challenges in identifying HER2-low breast cancer by IHC

Until recently, the focus of HER2 testing in pathology practice has been on making the binary distinction between HER2-positive and HER2-negative tumours. However, now that patients with HER2-low breast cancer are eligible for targeted therapy, tumour HER2 expression must be assessed and reported in a 3-tier rather than a 2-tier system. Today, it is essential to discriminate among HER2-positive breast cancer, HER2-low breast cancer, and breast cancer with a tumour HER2 IHC score of 0, which would not be eligible for treatment with either a traditional anti-HER2 monoclonal antibody or a HER2-based ADC.

Accurately and reproducibly distinguishing HER2-low from HER2 0 breast cancers is challenging. The standard practice for evaluating and quantifying HER2 IHC in routine practice is manual annotation,^11^ which is observer dependent and liable to subjective error. The prevalence of breast cancer means that its diagnostic management is likely to be carried out not only by specialists but also by general pathologists. Significant diagnostic variability has been reported among pathologists even in making the binary distinction between HER2-positive and HER2-negative tumours.^5^ The problem is particularly acute in relation to the distinction between IHC 1+ (incomplete membrane staining that is faint or barely perceptible and within >10% of the invasive tumour cells) and IHC 0 (no staining or membrane staining that is incomplete and is faint or barely perceptible and within ≤10% of the invasive tumour cells).^12^, ^13^, ^14^ Here, the weakness of the labelling means there is a greater potential for subjectivity than is the case for the distinction between IHC 2+ and 3+.

Along with the semi-quantitative and subjective nature of HER2 IHC assessment, heterogeneous HER2 expression within the tumour cell population can be another source of scoring variability.^5^^,^^15^^,^^16^ Again, this can have a disproportionate confounding effect on the accuracy and reproducibility of IHC scoring in HER2-low tumours.^17^

## The opportunity for digital technology to facilitate the identification of HER2-low breast cancer

Digital technology, such as automated tissue processing and staining, and digital data processing, storing, and management, has been used in the field of diagnostic breast pathology and IHC for decades.^11^ More recent applications of digital technology include whole-slide imaging (WSI), which is the process of digitising histopathology, IHC, or cytology slides using whole-slide scanners,^18^ and quantitative image analysis (QIA), which can be used to acquire quantitative and meaningful information from digital images.^19^ The process of transforming histopathology slides into digital images and their subsequent analysis and interpretation can be referred to as digital pathology.^20^^,^^21^ Digital pathology can aid pathologists in providing more quantitative, detailed, objective, and reproducible assessments of tissue biomarkers.^22^ Breast cancer IHC biomarkers, including HER2, were the first biomarkers to be analysed using QIA,^11^ and HER2 IHC scoring is one of the most frequent applications of QIA in clinical practice.^22^ QIA has been shown to provide superior reproducibility in HER2 scoring compared with manual assessment.^23^

One notable feature of WSI is that it provides a foundation for the application of artificial intelligence (AI) in digital pathology.^24^, ^25^, ^26^ The extraction of information from digitised pathology images using AI methods is a particular application of what has been termed computational pathology (CPATH).^27^ Current CPATH applications relate mainly to image analysis to improve the accuracy and reproducibility of variables traditionally assessed manually by the pathologist.^26^

Automated quantification of IHC staining intensity was an early application of statistical learning methods in digital breast cancer pathology,^28^ and CPATH with digital image analysis and AI-based methods holds great promise for efficient and accurate pattern recognition and quantification of HER2 IHC.^11^

QIA usually represents methods that attempt to represent and digitise images by statistically robust image feature representation accurately. A typical QIA tool usually limits the analysis to specific region of interest (ROI). Those ROIs are often manually selected by a pathologist. In AI powered CPATH, the algorithms can automatically detect and analyse an entire stained slide, involving not only tissue and/or cellular classification, target stain detection, segmentation, and stain quantification, but also enabling feature extraction, pattern recognition, and prediction.

AI-powered HER2 interpretation was shown to help reduce interobserver variability in scoring HER2 expression in a study in which 3 pathologists evaluated 209 whole-slide images first without and then with assistance from AI (Lunit SCOPE HER2). The use of AI improved concordance from 49.3% (103/209; Fleiss kappa 0.512) to 74.6% (156/209; Fleiss kappa 0.762) overall. The benefit of using AI was particularly notable in the scoring of HER2 IHC 1+ tumours, where it improved concordance from 25.7% (9/35; Fleiss kappa 0.242) to 68.9% (42/61; Fleiss kappa 0.687).^29^

Similar findings were reported from a study that explored the role of AI specifically in the interpretation of HER2 IHC scores of 0 and 1+.^30^ Fifteen pathologists evaluated 246 HER2 IHC slides without the assistance of AI and then again with AI assistance. Use of the AI algorithm improved interpretation accuracy from 0.80 to 0.93, as well as improving total consistency (intraclass correlation coefficient of 0.542 without AI vs. 0.812 with AI).^30^ Again, the improvement in consistency was particularly marked in the case of HER2 IHC 1+ tumours. Notably, the study showed that the use of AI can help to mitigate the effect of heterogeneity in tumour HER2 expression. The accuracy of pathologist-reviewed results in cases with heterogeneous staining was extremely poor (0.68) but was improved considerably (0.92) with the assistance of AI.

In addition to being used to quantify IHC expression objectively and accurately, CPATH can help in optimising the pre-analytical factors that can affect the performance of IHC.^11^ The staining consistency can be impacted by many pre-analytical factors from tissue preparation (tissue fixation, grossing, tissue processing, and embedding) or staining process (staining instruments, reagents, staining runs, and laboratories). CPATH algorithms can help normalise the staining quality and consistency. Stain normalisation is not strictly always powered by sophisticated AI models, although it is increasingly becoming the norm to use generative adversarial networks (GANs) for stain normalisation. Controlling variability in IHC staining quality is essential for accurate and reproducible quantification of HER2 expression using imaging analysis.^22^ AI performs better than eyeballing human assessment to ensure staining quality through comparison with well-characterised staining controls. AI tools can identify the spectrum of error on the scanned slides in terms of staining quality and even the quality of tissue fixation.^20^ AI-based image analysis can also be used to predict the degree of heterogeneity in the core biopsy that may necessitate repeat staining.^11^^,^^31^ A further opportunity for quality assurance afforded by AI-based tools results from the ability to predict marker expression from digitalised images of haematoxylin and eosin-stained slides.^11^^,^^32^

## Challenges in integrating CPATH in practice

While it has the potential to improve the accuracy and reproducibility of HER2 IHC scoring by pathologists, the adoption of CPATH by the pathology community has been relatively limited so far.^5^^,^^27^ This is despite evidence that a majority of pathologists are favourably disposed to the prospect of using it.^33^ Integrating CPATH into routine clinical practice involves navigating a number of technical, logistical, regulatory, and financial considerations,^26^ the details of which are beyond the scope of this review, but which have been extensively covered elsewhere.^21^^,^^25^^,^^27^^,^^34^^,^^35^ In the case of most models, these include the reliance on annotated training data, the limited availability of which can be a constraint and is something the community is trying to mitigate.^36^

At a basic level, the penetration of digital pathology into routine practice itself remains low,^11^ and many laboratories may simply lack the hardware necessary to begin considering CPATH. Not every laboratory has a digital scanner, for example.

Technical considerations can present obstacles to the adoption of CPATH. For example, the IHC quality criteria on which an algorithm depends may be so highly stringent as to make it impractical for routine clinical use, given the variation in staining intensity that can exist among different instruments, reagents, and staining runs.^22^ Interoperability can also be a barrier, for example through uncertainty over how algorithms perform across images from different scanners.

Then, there is the question of whether/how the potential for clinically relevant benefits can be reconciled with the substantial upfront investment required.^21^^,^^26^

There may be uncertainty over precisely what business/clinical case to identify. Laboratories are likely to adopt CPATH once they know which algorithm to choose for a given question (e.g., to diagnose a specific pathology or interpret a particular marker) and this is of relevance to a sufficient number of pathologies/markers to make it practical (e.g., programmed cell death ligand 1 [PD-L1] across tumour types with HER2/ER/PR in the breast plus Ki67 and counting of mitoses across tumour types).

An increasingly wide range of CPATH tools has become available in the HER2 space in recent years, making it difficult for pathologists to make a choice. While none of these has demonstrated superiority, performance would not necessarily be consistent in different laboratories in any case, nor would the tool necessarily be compatible with the majority of learning management systems or information management systems. Until there is a tool that meets these conditions, there will continue to be constraints on the adoption of CPATH.

## Summary

Developments in the armamentarium for the management of patients with breast cancer mean that today, it is essential in clinical practice to be able to discriminate precisely among HER2-positive, HER2-low, and HER2 IHC 0 tumours.

Manual, semi-quantitative evaluation of HER2 IHC staining is subject to substantial interobserver variability, not helped by the intratumoral heterogeneity of HER2 expression seen in breast cancer. Distinguishing IHC 1+ from IHC 0 tumours is more challenging than distinguishing between IHC 2+ and 3+ tumours, and heterogeneity is higher in HER2-low than in IHC 3+ tumours.

The recent confluence of WSI and AI-based technology has the potential to support pathologists in overcoming these challenges to achieve more accurate and reproducible HER2 IHC scoring. The deployment of CPATH is very likely to change the way pathology is practised,^21^ among other things strengthening its ability to meet the needs of precision medicine.^20^ A combination of AI and pathologists can yield results that are more accurate, consistent, timely, and useful beyond a human’s ability.^20^^,^^28^

AI algorithms are not yet widely used in the clinical setting for the identification of HER2-low breast cancer, and further research is required to refine them.^37^ Nevertheless, change is imminent, and despite current uncertainties and challenges, laboratories are expected to benefit from preparing for this sooner rather than later.^21^

## Financial support and sponsorship

10.13039/100004325AstraZeneca funded medical writing and editorial assistance, which was provided by David Cooke at Indigo Medical.

## Declaration of Competing Interest

The authors declare that they have no known competing financial interests or personal relationships that could have appeared to influence the work reported in this paper.
